# Suppression of droplets freezing on glass surfaces on which antifreeze polypeptides are adhered by a silane coupling agent

**DOI:** 10.1371/journal.pone.0204686

**Published:** 2018-10-05

**Authors:** Kazuya Koshio, Kazuhide Arai, Tomonori Waku, Peter W. Wilson, Yoshimichi Hagiwara

**Affiliations:** 1 Division of Mechanophysics, Graduate School of Science and Technology, Kyoto Institute of Technology, Matsugasaki, Sakyo-ku, Kyoto, Japan; 2 Faculty of Molecular Chemistry and Engineering, Kyoto Institute of Technology, Matsugasaki, Sakyo-ku, Kyoto, Japan; 3 Institute for Marine and Antarctic Studies, University of Tasmania, 20 Castray Esplanade, Hobart, Australia and Honors College, University of South Florida, Tampa, FL, United States of America; 4 Faculty of Mechanical Engineering, Kyoto Institute of Technology, Matsugasaki, Sakyo-ku, Kyoto, Japan; Institute of Materials Science, GERMANY

## Abstract

The development of ice-phobic, glass-substrate surfaces is important for many reasons such as poor visibility through the ice-covered windshields of vehicles. The present authors have developed new glass surfaces coated with a silane coupling agent and polypeptides whose amino-acid sequence is identical to a partial sequence of winter flounder antifreeze protein. We have conducted experiments on the freezing of sessile water droplets on the glass surfaces, and measured the droplet temperature, contact angle, contact area and surface roughness. The results show that the supercooling temperature decreased noticeably in the case where a higher concentration solution of polypeptide was used for the coating. The adhesion strength of frozen droplets was lowest in the same case. In addition, we observed many nanoscale humps on the coated surface, which were formed by polypeptide aggregates in the solution. We argue that the combination of the hydrophilic humps and the hydrophobic base surfaces causes water molecules adjacent to the surfaces to have a variety of orientations in that plane, even after the ice layer started to grow. This then induces a misfit of water-molecule spacing in the ice layers and consequent formation of fragile polycrystalline structure. This explains the lower values of ice adhesion strength and supercooling enhancement in the cases of the polypeptide-coated glass plates.

## Introduction

The development of ice-phobic surfaces is of importance because ice-covered surfaces often cause serious issues, such as (1) poor visibility through the windshields of aircraft, trains and automobiles; (2) poor visibility of traffic lights in snowy winter weather, (3) the breaking of power transmission lines; (4) deterioration of the aerodynamic performance of aircraft wings; and (5) damage to the casing of jet engines and air-conditioning equipment, to name just a few.

Various ice-phobic surfaces have been used in recent studies [1–6]. The types of these surfaces can be categorized as: hydrophilic, hydrophobic, super-hydrophobic (or textured) and finally, lubricant-infused [7]. Delays in freezing time (often called lag time) have been reported in the cases of the hydrophobic and super-hydrophobic surfaces [5]. A lowering of the strength of ice adhesion has been reported for all the surfaces except for the hydrophilic surfaces [4, 6, 8, 9]. Despite these efforts, a suitable surface treatment, which is necessary for reducing the aforementioned issues, has not yet been found, one which is durable and readily made.

With regard to ice growth on glass, antifreeze protein (AFP) from ocean pout (molecular weight, *M*_w_: 7 kDa [10]) and AFP from snow flea (*M*_w_: 13 kDa [11]) bound on glass surfaces were investigated [12]. In Ref. [12], significant delay in the freezing of condensed water was measured. A lowering of the supercooling temperature was obtained for AFP from *Chaetoceros neogracile* [13] (*M*_w_: 29.4 kDa [14]) and its mutant bound on aluminum surfaces. On the other hand, a raising of supercooling temperature was measured for AFP from the Antarctic marine diatom [15] (*M*_w_: 7 kDa). Although these AFPs may be promising, their thermal denaturation is inevitable. The temperature, *T*_d_, over which the thermal denaturation occurs, can be estimated from the following empirical relationship between *T*_d_ and *M*_w_ [16]; *T*_d_ [°C] = -2.1*M*_w_ [kDa] + 60. This relationship was derived from the experimental results for the solutions of hyperactive AFP (*M*_w_: 17 kDa) [17] and snailfish AFPs (*M*_w_: 9.3−9.6 kDa) [18]. The value of *T*_d_ for AFP from snow flea is approximately 33°C and the value of *T*_d_ for AFP from *Chaetoceros neogracile* is lower than 0 degrees. Thus, the thermal denaturation occurs during the AFP coating and before the freezing of condensation droplets on the AFP coated surfaces. It is therefore necessary to discover new AFP alternatives, which have higher value of *T*_d_, available for ice-phobic surface coating.

The present authors have focused on three polypeptides, synthesized by Kun and Mastai [19] based on segments of winter flounder antifreeze protein, as the alternatives. Kun and Mastai analyzed the secondary structure of these polypeptides by using circular dichroism spectroscopy, and the ice crystal morphology by using X-ray diffraction. Kun and Mastai also measured the thermal hysteresis (the difference between the melting point and freezing point) using an osmometer, and discovered that one of the polypeptides shows the thermal hysteresis being approximately 60% of the native protein hysteresis. The present authors expect that the denaturation of this polypeptide is unlikely. This is partly because the value of *T*_d_ for the polypeptide (*M*_w_: 1.046 kDa [20]) is 58°C, and partly because we have previously shown that a circular dichroism spectrum of the short-time preheated solution of the polypeptide was approximately the same as that of the unheated solution [20]. In addition, we have conducted experiments on unidirectional freezing and obtained the results in which the temperature at the ice/solution interface in the case of the preheated solution was lower than that in the case of unheated solution [20]. The short helical structure of the polypeptide, which includes eight hydrophobic amino-acid residues (seven Alanine and one Leucine), is maintained with strong hydrophobic interaction and hydrogen bonds. Thus, this polypeptide seems more promising to produce ice-phobic surfaces. However, no one has yet studied changes in supercooling temperature and ice adhesion strength for the polypeptide-coated surfaces.

In this study, we focus on reducing the issues mentioned above and on producing glass surfaces coated with the polypeptide by using a coupling agent and a linker. We conduct measurements on supercooling temperature and surface adhesion strength for freezing water droplets on these coated surfaces. Furthermore, with an atomic force microscope (AFM), we observe the polypeptide-bound surfaces both dried and droplet-deposited. The effects of polypeptide binding to the surface are also discussed. The present study represents an example of the creation of bio-inspired ice-phobic surfaces.

## Materials and methods

### Materials

The following reagents were purchased and used: 3-Aminopropyltrimethoxysilane (APTMS) (>97%, Wako Pure Chemical Industries, Ltd.), a 20% solution of glutaraldehyde (GA) (Wako Pure Chemical Industries, Ltd.) and a pH buffer solution of the mixture of sodium hydrogen carbonate and sodium hydroxide (pH = 9.6) (Tokyo Chemical Industry Co., Ltd.).

The sequence of amino-acid residues for the polypeptide used in this study is as follows: NH_2_—DTASDAAAAAAL—CONH_2_ (A: Alanine, D: Aspartic acid, L: Leucine, S: Serine, T: Threonine). This sequence and the terminuses are the same as those of the polypeptide mentioned in the Introduction. We purchased the synthetic polypeptide from GenScript Inc. (Taito, Tokyo, Japan).

### Polypeptide coatings

We used borosilicate glass plates of 15 × 15 × 0.15 mm^3^ as substrates. We measured the surface roughness of the glass plates with AFM. The profile roughness parameter and its standard deviation were 0.71 nm and 0.26 nm, respectively. We conducted the following procedures for the polypeptide coating to the substrates.

First, the hydrolysable group of APTMS was bound to the glass surface. For this purpose, the glass plates were soaked in the APTMS solution for three hours. It is known that it takes approximately several hours to complete the hydrolysis of APTMS. We had already confirmed that the deviation of the contact angle for the APTMS-coated glass surfaces in the case of 3-hour soaking was the minimum among a 1-hour to 24-hour period of soaking [21] (See S1 Fig). After that, the plates were washed with both DI water and ethanol, and dried in an oven at 100°C for one hour. The APTMS solution was produced by dropping APTMS gradually into an ethanol solution of 2% while stirring for 30 min.

Secondly, an aldehyde group of GA was bound to the organo-functional group of APTMS. To produce a 2% solution of GA, the pH buffer solution and the GA solution were mixed in a tube by using a stirrer for 30–60 min. The APTMS-bound glass plates were soaked in this GA solution in the oven at 37°C for two hours. After that, the plates were washed with DI water.

Thirdly, the antifreeze polypeptide was bound to another aldehyde group of the GA. For this purpose, the polypeptide was dissolved into the pH buffer solution, pipetting was done for the complete dissolution, and the solution was dripped onto the GA-coated surface. The concentration of polypeptide used was both 0.1 and 0.5 μMol. Finally, the surface was dried in the oven at 37°C for two hours.

**Fig 1:**
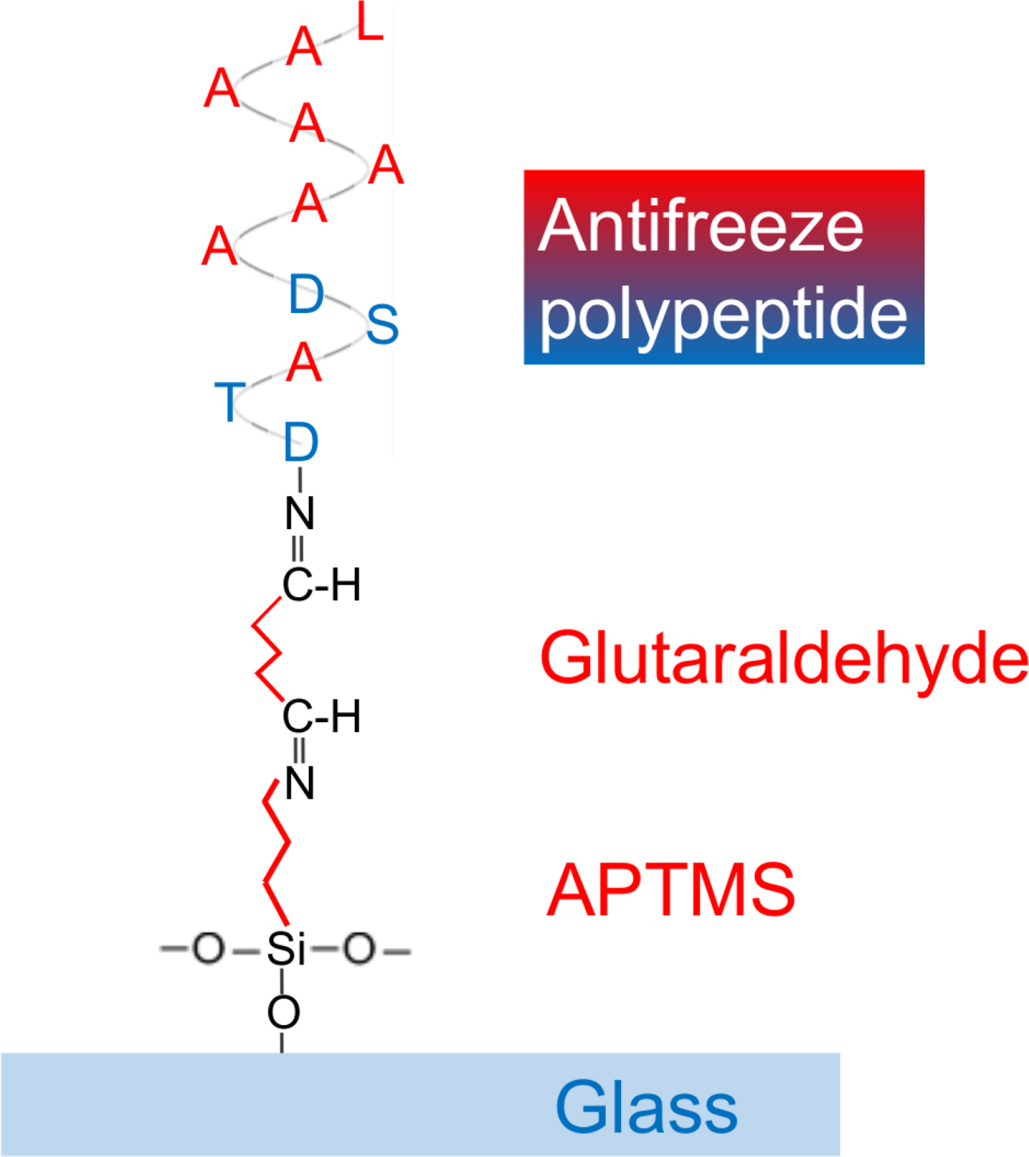
Bindings of the materials. Hydrophobic parts are shown in red; the Alanine and Leucine residues in the polypeptide, and the hydrocarbon chains in GA and APTMS. Hydrophilic parts are shown in blue; the Aspartic acid, Threonine and Serine residues in the polypeptide, and the glass.

As a result of the coating, four hydrophilic amino-acid residues (two Aspartic acid, one Threonine and one Serine) faced the GA because the N-terminus of the polypeptide was expected to be bound to the GA (See Fig 1). Thus, seven hydrophobic amino-acid residues (six Alanine and one Leucine) were expected to face the air and the droplets. Glass plates bound with only APTMS and unprocessed glass plates were used to obtain reference results.

When we measured the ice adhesion strength, the coating areas of the polypeptide were identical. For this purpose, the polypeptide solution was dropped inside a polyacetal washer (8.0 mm in inner diameter, 10 mm in outer diameter and 5.0 mm in thickness) put on each of the test plates. The reason for the area restriction is to obtain the binding of the polypeptide to the GA on the identical area repeatedly.

### Droplet freezing measurement

Each of the glass plates was set on the rectangular protrusion (50mm × 46mm × 3mm) from a copper plate (100mm × 100mm × 3mm). This copper plate was screwed and cooled with a Peltier device with a coolant flowing through the device (Sensor Controls Co., Ltd., DET-4120). The coolant flowrate and the input voltage to the device were controlled with a programmable controller (Sensor Controls Co., Ltd., FC3510) to adjust the temperature measured with a thermocouple inside the device to the predetermined temperature at any instant. The accuracy of the controller was ±0.1°C.

A DI water droplet 10μL in volume was placed on each of the glass plates using a micropipette. After the droplet placement, the Peltier device controller was operated to obtain the following cooling condition: (1) the predetermined temperature was maintained at 5°C for the first 5 min, (2) then the predetermined temperature was decreased at a constant rate of -2°C/min.

Successive images of the freezing droplet were captured with a video microscope with an LED light source (Shodensha, Japan, TG70TV). The pixel resolution of the video microscope was 14.5μm × 14.5μm. The top view or side view of the sessile droplet was captured for 25 min. At least five runs were carried out for each of the different plate surfaces. In each run, a new plate and fresh DI water were used. The apparatus was installed in a temperature-controlled room at 5°C. The relative humidity was approximately 40%.

We also measured the temperature inside the freezing droplets while capturing the images. A type K thermocouple of 0.1mm in diameter was horizontally inserted into near the center of the droplets. The volume of the thermocouple in the droplet was approximately 1.9% of the droplet volume. Similar temperature measurements with fine thermocouples were conducted for the freezing droplets 21 μl [22] and 10 μl [23] in volume. Thus, it is surmised that the thermocouple did not cause a significant attenuation of the ice growth.

### Measurements of contact angle and contact area

We captured the front-view images of droplets 3μL in volume on the surfaces using the video microscope. We measured the contact angle by processing the captured images using ImageJ software.

We also captured the top-view images of these droplets on the surfaces using the video microscope. We measured the contact area of the droplets by counting the pixels covering the wetted area in the processed images.

### Ice adhesion strength measurement

**Fig 2:**
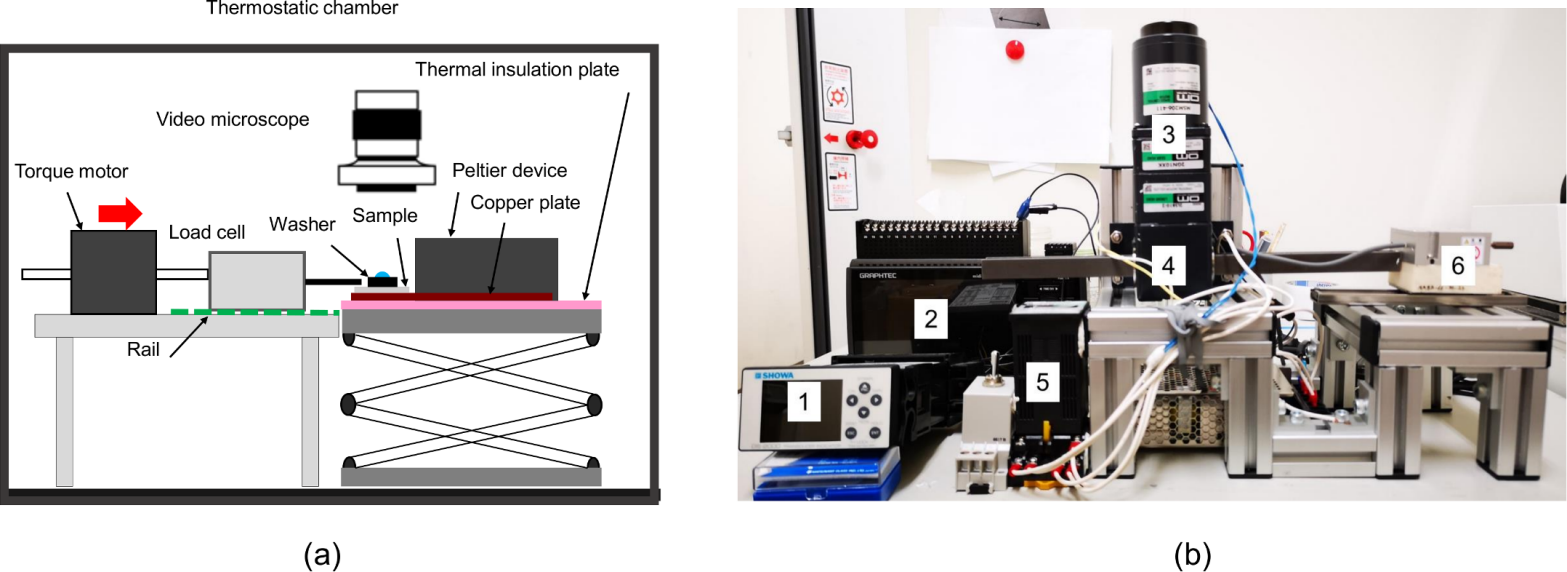
Apparatus for ice adhesion strength measurement. (a) Schematic diagram, (b) Main part of apparatus. 1; a digital transducer, 2; a data logger, 3; a torque motor, 4; a linear head of the motor, 5; a motor-speed controller, 6; a load cell.

Fig 2 shows the schematic of the apparatus for ice adhesion strength measurement. This apparatus consists of a torque motor, a rail, a load cell, the video microscope, the cooling device and a jack.

The experiment procedures are as follows: First, each of the glass plates was placed on the rectangular protrusion (30mm × 30mm × 3mm) from a copper plate (100mm × 100mm × 3mm). The surface of the protrusion was coated with heat conduction grease. Four fixtures, consisted of screws and washers, were used for fixing the glass plate on the protrusion completely. The copper plate was screwed and cooled with the aforementioned Peltier device. After checking that the glass-plate surface temperature, measured with two thermocouples attached on the glass-plate surface, was stable within a range of 4–6°C, a steel washer (S45C, 3.0 mm in inner diameter, 5.0 mm in outer diameter and 2.0 mm in thickness) was placed on the glass-plate surface. As a result, the contact area of ice was 7.1 mm^2^, and this contact area was circular. The contact area is much smaller than the square contact areas of ice realized with cuvettes placed on cooling superhydrophobic surfaces in the measurements of ice adhesion strength [8, 24–28].

Secondly, DI water 10 μL in volume was deposited inside the washer using a micropipette. It was confirmed with the microscope that there was no leak of water from below the washer. After that, the cooling device was operated with the same cooling rate (-2°C/min.) as that for the freezing measurement mentioned above until the device temperature reached -20°C. Then, the device temperature was maintained at -20°C to attenuate the change in the droplet temperature with time, which was a different device operation from that for the freezing measurement. Since it is difficult to insert a thermocouple into a droplet through the washer, we pasted a fine type K thermocouple on each plate surface outside the washer. The ice adhesion strength measurement mentioned above was conducted in periods of approximately 15 min. through which the surface temperature changed from -15°C to -17°C. Although this temperature range was higher than the supercooled temperature in the freezing measurement, the droplets completely froze. This is because the cooled washer slightly enhanced the droplet freezing.

Thirdly, the torque motor was driven for its head to push one side of the load cell, and consequently a bar attached to the other side of the load cell pushed the washer. The push speed was set at 0.08 mm/s. This is the lowest speed with the guarantee of the accuracy of ±5% for the preset speed. The push speed is lower than that in references [8, 24–29]. The vertical position of the washer was adjusted using the jack so that only the force in the horizontal direction acted on the washer without any rotational moment.

The time change in the reaction force obtained from the load cell was measured. The reaction force increased with time. This dependency is categorized into two types; linear (or elastic) and nonlinear (or viscoelastic). The former type (hereafter called type A) is a result of adhesion failure occurring from the separation of ice from the glass-plate surface. The latter type (hereafter called type B) is a result of cohesion failure occurring from the separation of ice inside crystals [30]. In the latter case, part of the ice remains on the glass-plate surface. In this report, we did not take account of the type-B reaction force in the calculation of ice adhesion strength. The maximum value of the type-A reaction force divided by the contact area was defined as the ice adhesion strength.

## Results and discussion

### Changes in droplet temperature

**Fig 3:**
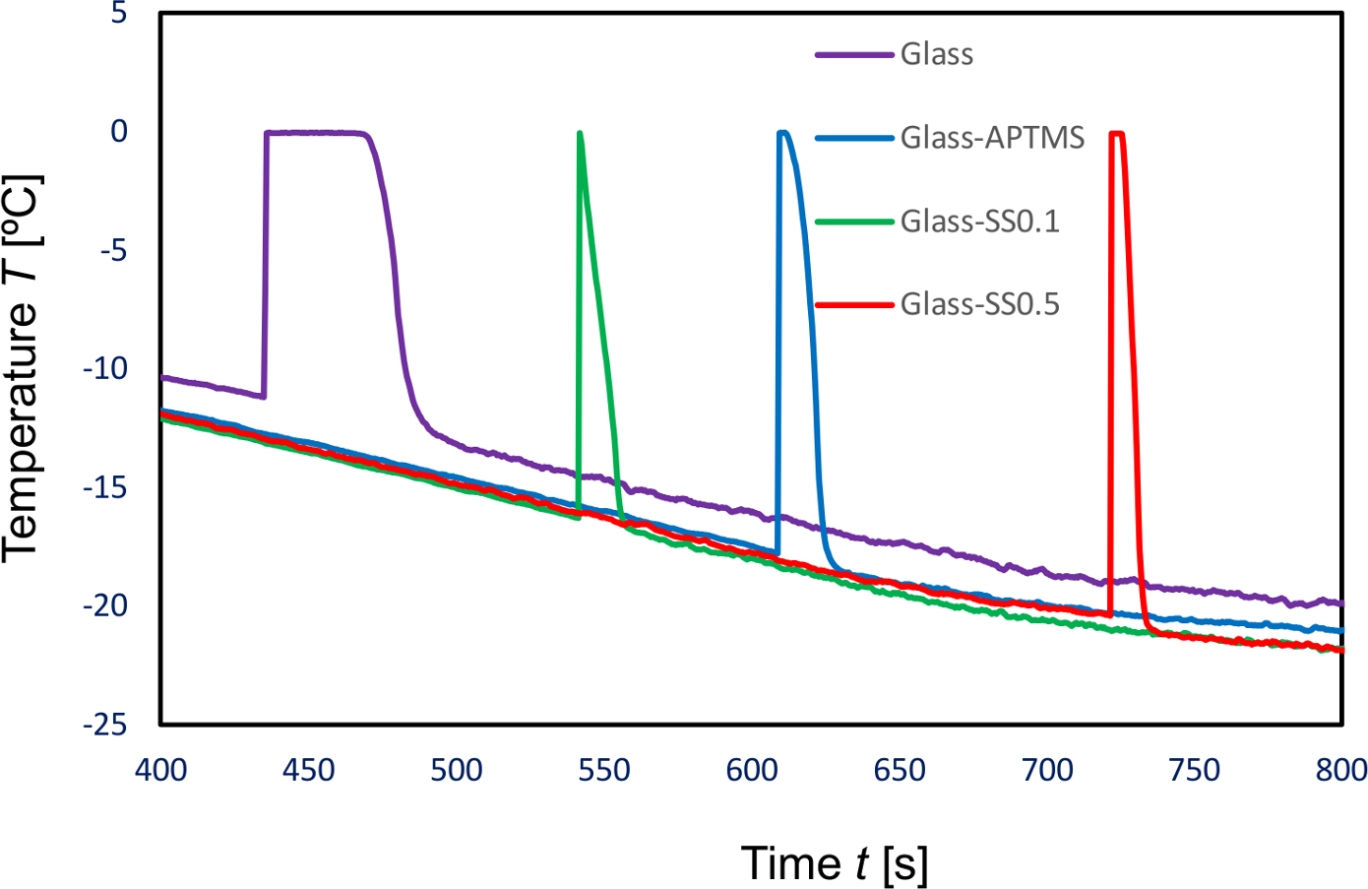
Typical examples of cooling curves. SS0.1 and SS0.5 stand for the cases with the polypeptide solutions of 0.1 and 0.5 μMol, respectively.

Fig 3 shows typical examples of cooling curves, i.e. time vs changes in the temperature of water droplets. The size of the glass plates was 15 × 15 × 0.15 mm^3^. The origin of time in this figure was taken as the temperature measured with the thermocouple inside the Peltier cooling device was at 0°C. In the case of unprocessed glass surfaces, the droplet temperature decreased with time until 435s. The rate of the temperature decrease is approximately the same as the predetermined rate of temperature decrease for the cooling device. Then, the temperature abruptly rose due to the elimination of the supercooled state, i.e. nucleation and freezing. Just before this increase in the temperature, the droplet became opaque. This was a result of the rapid developing of an ice shell along the droplet surface. This ice shell was proved by blowing water off with air by a manual blower. The ice shell originated from the contact line (air-water-glass interface) by frost crystals, which grew on the area adjacent to the droplet on the surface. Similar production of ice shells as a result of frost crystals was observed by Oberli et al [31].

The growth of the ice shell was followed by the growth of an ice layer from the contact area of the droplet. In a region near the water/ice-layer interface, the temperature was approximately 0°C because the latent heat of fusion was transferred by conduction through the ice layer to the cooling plates. This is the reason for the approximate 0°C being measured with the thermocouple inside the droplets for short periods. This temperature is taken to be the melting point. When the droplet completely froze, the temperature dropped rapidly because the heat conduction inside the frozen droplet was predominant. Eventually, the decreasing rate of temperature became similar to the predetermined rate.

It is worth noting that the ice shell originated also from the top of droplets occasionally. In this case, the frost crystals did not grow on the area adjacent to the droplet on the glass-plate surface. It is probable that the frost crystals generated around the top of droplets. This frost generation was able to occur when the local concentration of water vapor around the top of droplets was higher than that near glass-plate surfaces.

In the case of the surfaces coated with APTMS and polypeptide, supercooling was retained more than 100 seconds longer on average compared with the APTMS-coated surfaces. Table 1 shows comparisons of the temperature just before its rise (hereafter called the supercooling temperature), and the melting point. The melting point decreased slightly as a result of the coating. In contrast with this, the supercooling temperature dropped noticeably as a result of the coating, and depended on the coating materials as follows: (1) The supercooling temperature in the case of the APTMS-coated surface was lower than that in the case of the unprocessed surface, (2) The supercooling temperature rose slightly as a result of the polypeptide coating using the lower concentration solution, and (3) The supercooling temperature in the case of the polypeptide coating using the higher concentration solution was lower than that using the lower concentration solution. The reasons for these changes in the temperature will be discussed in the following sections.

**Table 1:** Comparison of the supercooling temperature and melting point.

	Glass	with APTMS	with APTMS and SS0.1	with APTMS and SS0.5
**Supercooling temp.** (°C)	-12.1 (0.83)	-17.9 (1.0)	-15.8 (1.6)	-18.4 (2.1)
**Melting point** (°C)	-0.07 (0.04)	-0.23 (0.17)	-0.20 (0.15)	-0.26 (0.18)

The values in the brackets show the standard deviation. SS0.1 and SS0.5 stand for the cases with the polypeptide solutions of 0.1 and 0.5 μMol, respectively.

### Contact angle and contact area

Table 2 shows the results for the contact angle and contact area. The size of the glass plates was 15 × 15 × 0.15 mm^3^. The contact angle of the APTMS-coated surfaces was higher than that of the unprocessed glass surfaces. This is due to the fact that the amino group of APTMS is less hydrophilic than an unprocessed glass surface. The average contact area for the APTMS-coated surfaces was approximately two-thirds of the contact area for the unprocessed surfaces [21]. This smaller area leads to lower heat removal from the droplets, and thus the attenuation of ice growth. In addition, the growth of frost crystals on the APTMS-coated surfaces was found to be slower than that on the unprocessed glass surfaces [32]. These two findings are the main reasons for the supercooling enhancement by the APTMS coating.

**Table 2:** Comparison of the contact angle and contact area.

	Glass	with APTMS	with APTMS and SS0.1	with APTMS and SS0.5
**Contact angle** (°)	24.5 (2.4)	70.7 (7.3)	78.0 (2.0)	59.6 (0.9)
**Contact area** (mm^2^)	7.36 (0.09)	4.76 (0.08)	4.09 (0.07)	5.75 (0.27)

The values in the brackets show the standard deviation. SS0.1 and SS0.5 stand for the cases with the polypeptide solutions of 0.1 and 0.5 μMol, respectively.

When the polypeptides were linked with APTMS on the surface, the contact angle increased in the case with the lower concentration solution, while it decreased in the case with the higher concentration solution. Nevertheless, the contact angles in these two cases were still much lower than those for almost all ice-phobic surfaces produced to date which are hydrophobic or super-hydrophobic.

### Surface roughness

**Fig 4:**
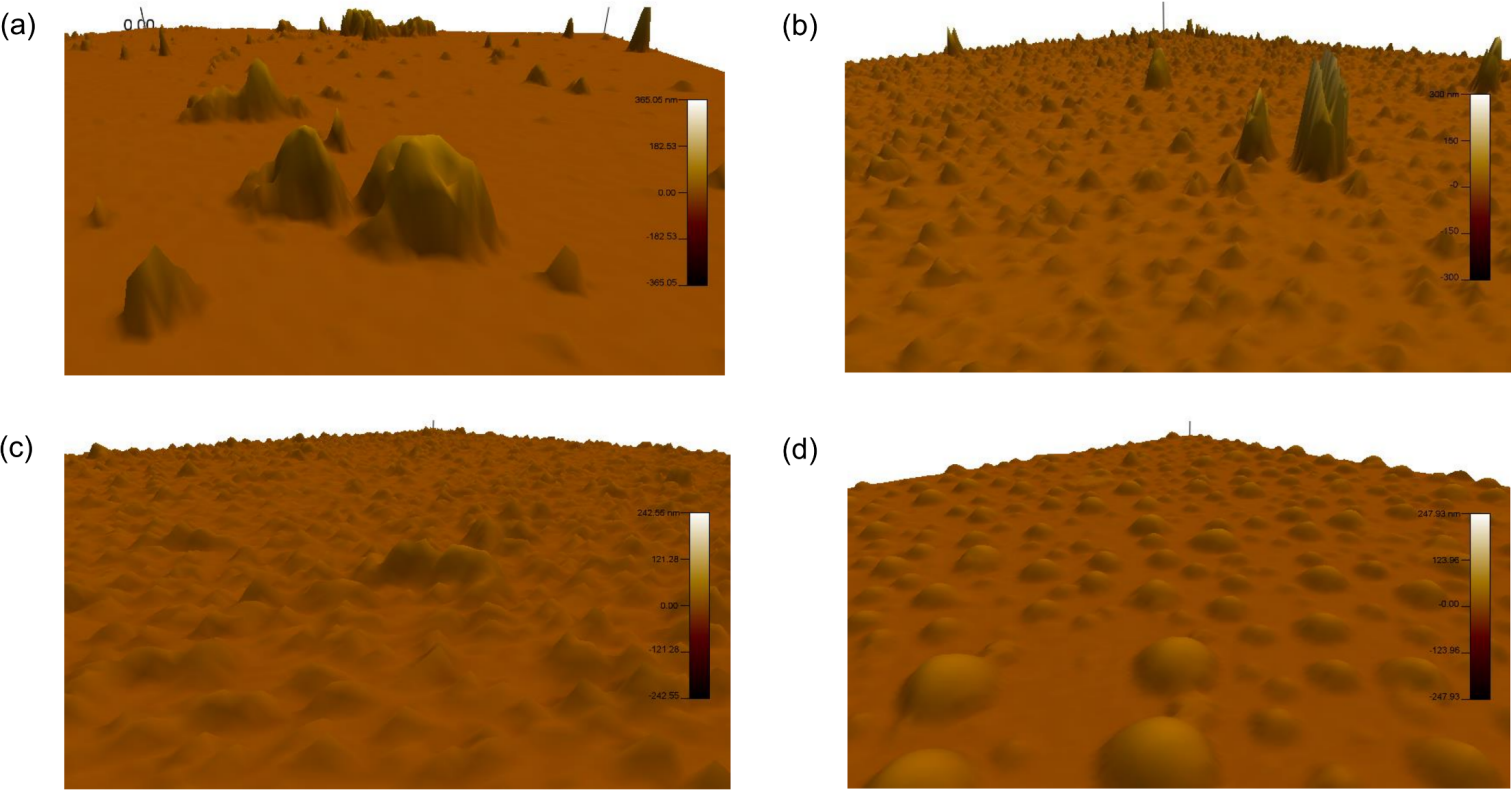
Typical examples of surface roughness. (a) A low resolution observation in the case with the polypeptide solutions of 0.1 μMol. An area of approximately 2.8 × 10^2^ μm^2^ is seen. (b) A low resolution observation in the case with the polypeptide solutions of 0.5 μMol. An area of approximately 3.0 × 10^2^ μm^2^ is seen. (c) A high resolution observation in the case with the polypeptide solutions of 0.1 μMol. An area of approximately 12 μm^2^ is seen. (d) A high resolution observation in the case with the polypeptide solutions of 0.5 μMol. An area of approximately 16 μm^2^ is seen.

To elucidate the difference in the surface wettability mentioned above, we measured the roughness of the polypeptide-coated surfaces by using an AFM (Asylum, MFP-3D Classic). We selected two observation areas; (1) an area of 400μm^2^ for low-resolution scanning, and (2) another area of 25 μm^2^ for high-resolution scanning. The larger area was used to detect large-scale roughness, while the smaller area was used to detect small-scale roughness. Fig 4 shows typical examples of the surface roughness. Several large humps are seen in the wider observation areas, regardless of the concentration of polypeptide solution, in Fig 4(A) and 4(B). On the other hand, many small humps are seen in the smaller observation areas, regardless of the polypeptide concentration, in Fig 4(C) and 4(D). Similar humps were not observed in the case of the APTMS-coated surface. Thus, the humps were produced by the polypeptide.

Hereafter, we will discuss the statistical quantities of the humps. Software incorporated in the AFM was used to obtain the peripheral length, bottom area, height and volume of humps, and the proportion of the hump area to the whole area. This proportion was estimated by the following procedure: First, we obtained the probability distribution of height from the result of a scanned surface. Secondly, we identified a specific height which gave an inflection point in the probability distribution. We adopted this specific height as a threshold to distinguish humps from the minute roughness of surfaces. Finally, we obtained the proportion from the number of points where the local height was higher than the threshold. Table 3 shows the proportions of areas of humps. It is found that the proportion increases with an increase in the concentration of the polypeptide solution, regardless of the size of the humps.

**Table 3:** Comparison of the dimensions of humps.

	with APTMS and SS0.1		with APTMS and SS0.5	
	Small humps	Large humps	Small humps	Large humps
**Proportion of hump area** (%)	12	0.98	25	4.1
**Peripheral length** (μm)	0.54 (0.28)	3.7 (1.3)	0.71 (0.08)	2.0 (0.4)
**Bottom area** (μm^2^)	0.012 (0.011)	0.54 (0.23)	0.037 (0.009)	0.30 (0.10)
**Height** (nm)	25 (9.5)	93 (26)	26 (2.2)	106 (21)
**Volume** (μm^3^)	2.0 × 10^−4^ (1.0×10^−4^)	0.020 (0.0067)	6.3 × 10^−4^ (1.7×10^−4^)	0.017 (0.011)
**Number of polypeptides** (× 10^6^)	0.074 (0.053)	0.77 (0.25)	0.24 (0.065)	0.64 (0.042)

The values in the brackets show the standard deviation. SS0.1 and SS0.5 stand for the cases with the polypeptide solutions of 0.1 and 0.5 μMol, respectively.

We manually determined the periphery of selected humps from the two-dimensional AFM results. The bottom area for each hump was automatically calculated from the periphery using the software. Table 3 shows the average values and standard deviations of the peripheries and bottom areas of five small humps and five large humps in the cases of two concentration conditions of polypeptide solutions. It is found that the average values of the periphery and bottom area for the small humps increase with an increase in the concentration of the polypeptide solution. On the other hand, the average values of the large humps decrease with an increase in this concentration. This inconsistency will be discussed below.

It is noted that the equivalent diameter of the bottom area of the selected small humps was in the range of 0.24–1.8 × 10^2^ nm in the case of the lower concentration of the polypeptide solution, and was in the range of 1.7–2.4 × 10^2^ nm in the case of the higher concentration of the polypeptide solution. These diameter ranges are within 0.20–3.6 × 10^2^ nm, which was measured from the images of aggregates in the polypeptide solution (0.96 μMol) by using a transmission electron microscope [20]. Thus, it can be concluded that the small humps were produced by the bonding of polypeptide aggregates. On the other hand, the equivalent diameter of the bottom area of the selected large humps was in the range of 4.4–9.9 × 10^2^ nm in the case of the lower concentration of the polypeptide solution, and was in the range of 4.9–7.8 × 10^2^ nm in the case of the higher concentration of the polypeptide solution. These diameter ranges are much higher than the diameter range of the aggregates mentioned above. Thus, the large humps were concluded to be produced by the accumulation of many aggregates during the polypeptide coating procedures.

The local height in a masking area including a hump was evaluated from the difference between the local surface and the lowest surface in the periphery. The lowest surface was slightly lower than the threshold mentioned in paragraph two of this section. The height of the hump was defined as the highest value of the local height in the masking area. Table 3 shows the height of the five small humps and five large humps in the cases of two concentration conditions of polypeptide solutions. The average height of the humps is found to slightly increase with an increase in the concentration of the polypeptide solution, regardless of the size of the humps.

The volume of a hump was calculated from the summation of the local heights over the bottom area of this hump. It is found from Table 3 that the average volume of the small humps increases with an increase in the concentration of the polypeptide solution, while the average volume of the large humps decreases with an increase in this concentration. This inconsistency is similar to that of the periphery and bottom area mentioned above. The inconsistencies occurred because the large humps in the case of low concentration of the polypeptide solution had three-dimensionally complex shapes with long peripheries and large bottom areas.

We calculated the numbers of polypeptides which the humps were composed of. For this purpose, we assumed that the volume of each polypeptide can be expressed with the volume of a cylindrical column in which all the atoms of the peptide are included. The axial length and diameter of the column were respectively estimated to be 1.7 nm and 1.0 nm from the atom location in the first 12 amino-acid residues of the winter-flounder AFP model in our previous molecular dynamic simulation [33]. In addition, we assumed a 0.15-nm spacing around the surface of each column to reduce the effects of repulsive forces and to allow hydrogen bonds among adjacent polypeptides. The average number of the column (i.e. polypeptide) was in the range of 0.074–0.24 × 10^6^ for small humps, and was in the range of 0.64–0.77 × 10^6^ for large humps. The total number of polypeptides contained in all the humps can be calculated from these number ranges, the bottom areas and the proportion of hump areas. The total number is expected to be much lower than the total number in the solution dropped on the glass plates. Thus, the majority of the polypeptides were either adhered on the base flat surfaces or dissolved into DI water for rinsing the surface of the plates.

The surface of each hump is expected to be hydrophilic because the hydrophilic amino-acid residues (Aspartic acid, Serine and Threonine) of the polypeptides tend to face the outside of the aggregates and the hydrophobic amino-acid residues of the polypeptides (Alanine and Leucine) tend to face the inside of the aggregates. Thus, the surfaces of the humps can be hydrophilic. This is consistent with the aforementioned result where there was a low contact angle in the case of the higher concentration of polypeptide solution. On the other hand, there are many flat areas where non-aggregated polypeptides are expected to bind to GA via the N-terminus. Consequently, seven hydrophobic amino-acid residues (six Alanine and one Leucine) of the polypeptide are exposed, and thus, these areas tend to be hydrophobic. This is consistent with the aforementioned result where there was a high contact angle in the case of the lower concentration of polypeptide solution.

We also measured the surface roughness of glass plates partially covered with a water droplet by using a liquid-specialized cantilever of the AFM. Similar humps were observed on the surfaces of these glass plates. Thus, the binding of the aggregates to APTMS molecules was not destroyed by water droplets.

### Ice adhesion strength

**Fig 5:**
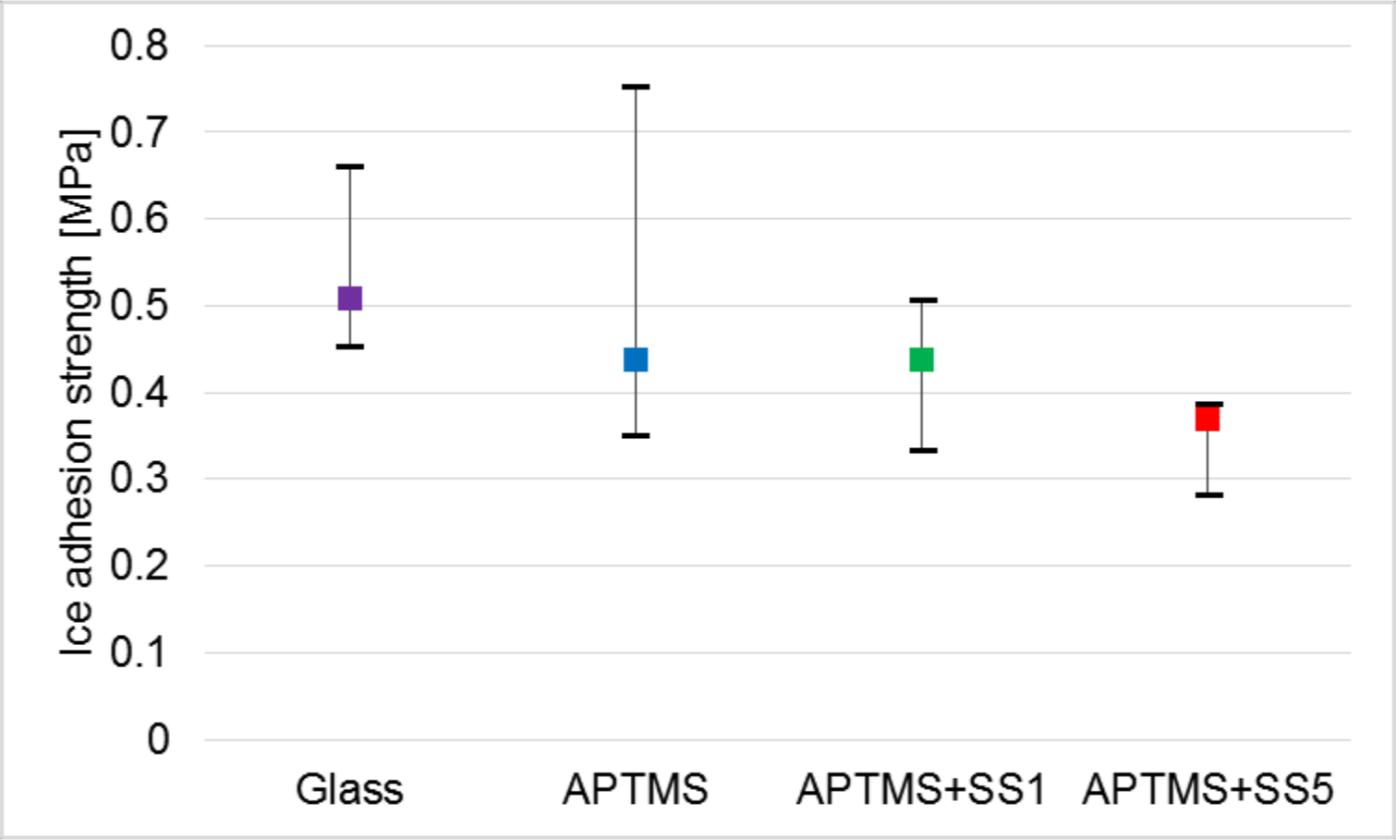
Ice adhesion strength. SS1 and SS5 stand for the cases with the polypeptide solutions of approximately 0.1 and 0.5 μMol, respectively. The average mass per unit area for each case is approximately half of that for the case of the other measurements due to the differences in the dropped volume and wetted area.

Fig 5 shows the ice adhesion strength for the glass-plate surfaces. The size of the glass plates was 15 × 15 × 0.15 mm^3^. The bars in this figure indicate the maximum and minimum values, and the symbols indicate the median values. The median value of the unprocessed glass surface is approximately 10% lower than the average strength measured at -18°C by Chernyy et al [27]. The median values in the cases of the APTMS-coated surfaces and GA-coated surfaces are lower than the median value of the unprocessed surface. In the cases of the polypeptide-coated surfaces, the values are equal to or lower than the value of the APTMS-coated surfaces or the unprocessed surfaces. Thus, the polypeptide-coated surfaces are effective for reducing the ice adhesion strength.

**Fig 6:**
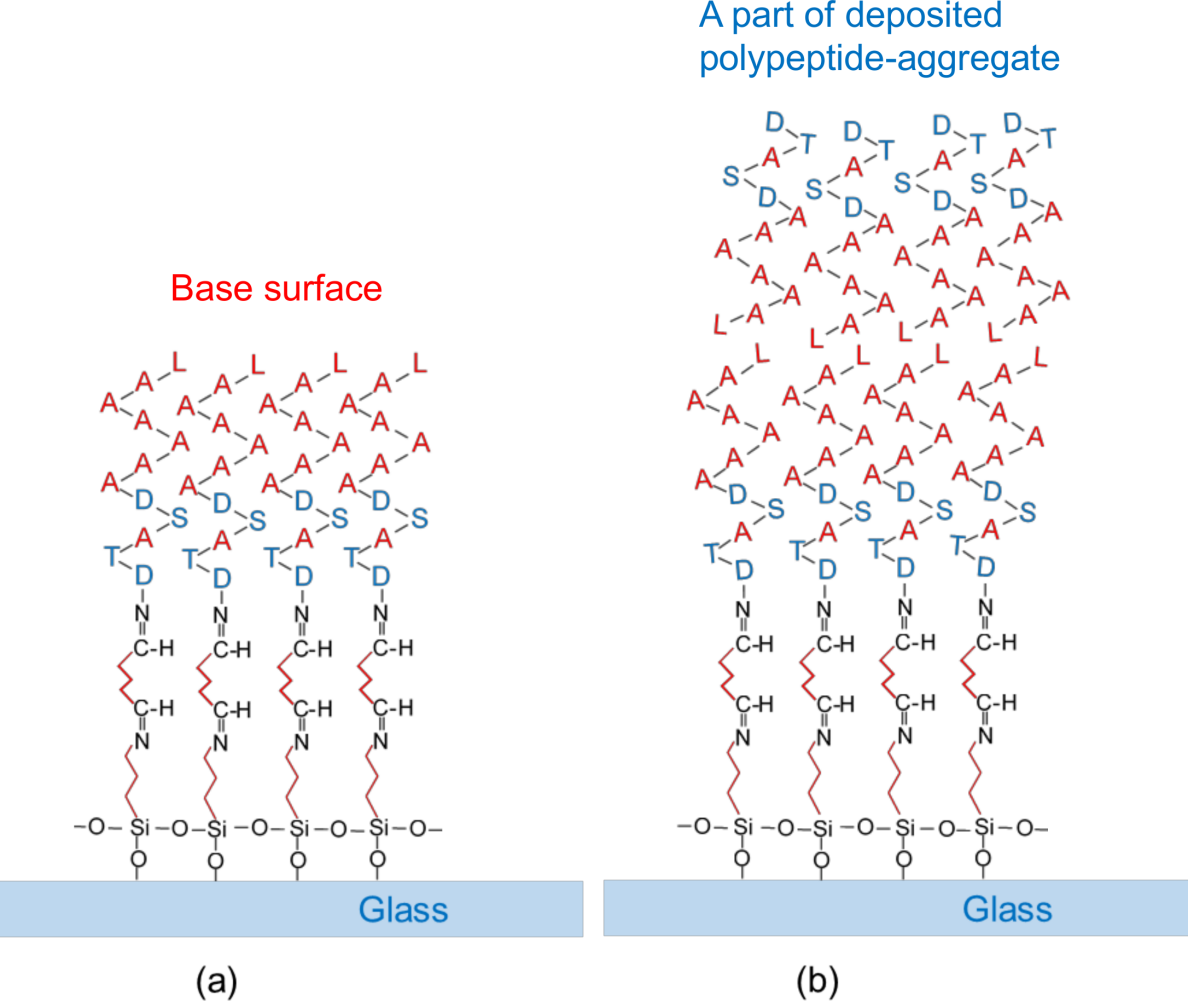
Two types of surfaces in the case of polypeptide coating. (a) Hydrophobic amino-acid residues (Alanine and Leucine) are exposed on the base surfaces, (b) Hydrophilic amino-acid residues (Aspartic acid, Threonine and Serine) are exposed on the surfaces of polypeptide aggregates.

The difference between the maximum and minimum values is seen to be large in the cases of the unprocessed surfaces and APTMS-coated surfaces. This is because the possibility is not low that the ice separation similar to the type B separation may occur (e.g., a thin ice layer remains on the surface as a result of the separation). On the other hand, the difference between the maximum and minimum values is small in the other two cases. This is because there is a very low possibility of the separation of ice similar to the type-B separation of ice occurring. The coexistence of (1) many hydrophilic, amino-acid residues exposed on the hump surfaces and (2) hydrophobic, ice-independent amino-acid residues exposed on the base surfaces (See Fig 6) may cause a variety of orientations of water molecules adjacent to the surfaces. This leads to the supercooling enhancement. Once the ice layer has been generated on the surfaces, the orientations of ice crystal axes differ locally, even though the basal plane of ice fundamentally faced the surface [34]. This causes a misfit of water-molecule spacing in the ice layers. As the ice layers grow, the degree of misfit increases, i.e., the degree of epitaxy of water molecules lowers. Thus, the formation of polycrystalline structure with the various direction of the crystal axis is enhanced. This polycrystalline structure is probably fragile. This is the reason for the lower values of ice adhesion strength and lower possibility of type-B-like separation in the case of the polypeptide-coated glass plates.

## Conclusions

We conducted experiments on the freezing of water droplets on cooled glass surfaces. The antifreeze polypeptides were coated to the surfaces with 3-Aminopropyltrimethoxysilane and a glutaraldehyde layer. The amino-acid residues of the polypeptides were identical to the twelve amino-acid residues of winder flounder antifreeze protein. The cooling rate of the electronic device was -2.0°C/min. We measured the droplet temperature, the contact angle, the contact area and the ice adhesion strength. It was found that the supercooling temperature in the cases coated with the polypeptides with the higher concentration solution was the lowest among all the glass plates. Also, the adhesion strength of frozen droplets in the glass plates coated with the polypeptides with the higher concentration solution was lower than that in almost all the other treatments used in the present study. In addition, it was found from the observation through AFM that many aggregates of the polypeptide were fixed on the surfaces as humps of various sizes. The combination of (1) hydrophilic humps due to the hydrophilic amino-acid residues of the polypeptides, and (2) hydrophobic base surfaces due to the hydrophobic amino-acid residues of the polypeptides causes various orientation of water molecules adjacent to the surfaces even after the ice layer started to grow. This leads to misfit of water-molecule spacing in the ice layers, and consequent formation of fragile polycrystalline structure. This is the reason for the lower values of ice adhesion strength, lower possibility of type-B-like separation and supercooling enhancement in the cases of the polypeptide-coated glass plates.

## Supporting information

S1 Fig Dependency of contact angle on soaking period for APTMS solution. (a) unprocessed glass plates, (b) in the case where the concentrations of APTMS and ethanol were 1 wt%, (c) in the case where the concentrations of APTMS and ethanol were 2 wt% and 1 wt%, respectively, and (d) in the case where the concentrations of APTMS and ethanol were 2 wt%.
(TIF)

## References

[1] Wang C, Fuller T, Zhang W, Wynne KJ. Thickness dependence of ice removal stress for a polydimethylsiloxane nanocomposite: Sylgard 184. Langmuir 2014; 30: 12819–12826. 10.1021/la5030444 25299447

[2] Jung S, Dorrestijn M, Raps D, Das A, Megaridis CM, Poulikakos D. Are superhydrophobic surfaces best for icephobicity? Langmuir 2011; 27: 3059–3066. 10.1021/la104762g 21319778

[3] Eberle P, Tiwari MK, Maitra T, Poulikakos D. Rational nanostructuring of surfaces for extraordinary icephobicity. Nanoscale 2014; 6: 4874–4881. 10.1039/c3nr06644d 24667802

[4] 隠すSubramanyam SB, Rykaczewski K, Varanasi KK. Ice adhesion on lubricant-impregnated textured surfaces. Langmuir 2013; 29: 13414–13418. 10.1021/la402456c 24070257

[5] Wilson PW, Lu W, Xu H, Kim P, Kreder MJ, Alvarenga J et al Inhibition of ice nucleation by slippery liquid-infused porous surfaces (SLIPS). Phys. Chem. Chem. Phys. 2013; 15: 581–585. 10.1039/c2cp43586a 23183624

[6] Chen J, Dou R, Cui D, Zhang Q, Zhang Y, Xu F et al Robust prototypical anti-icing coatings with a self-lubricating liquid water layer between ice and substrate. ACS Appl. Mater. Interfaces 2013; 5: 4026–4030. 10.1021/am401004t 23642212

[7] Kreder MJ, Alvarenga J, Kim P, Aizenberg J. Design of anti-icing surfaces: smooth, textured or slippery? Nature Reviews Materials 2016; 1: 1–15.

[8] Wang Y, Xue J, Wang Q, Chen Q, Ding J. Verification of icephobic/anti-icing properties of a superhydrophobic surface. ACS Appl. Mater. Interfaces 2013; 5: 3370–3381. 10.1021/am400429q 23537106

[9] Petrenko V F, Peng S. Reduction of ice adhesion to metal by using self-assembling monolayers (SAMs). Can. J. Phys 2003; 81: 387–393.

[10] Sönnichsen FD, DeLuca CI, Davies PL, Sykes BD. Refined solution structure of type III antifreeze protein: hydrophobic groups may be involved in the energetics of the protein–ice interaction. Structure. 1996; 4: 1325–1337. 8939756 10.1016/s0969-2126(96)00140-2

[11] Pentelute BL, Gates ZP, Tereshko V, Dashnau JL, Vanderkooi et al X-ray structure of snow flea antifreeze protein determined by racemic crystallization of synthetic protein enantiomers. J. Am. Chem. Soc. 2008; 130: 9695–9701. 10.1021/ja8013538 18598029 PMC2719301

[12] Esser-Kahn P, Trang V, Francis MB. Incorporation of antifreeze proteins into polymer coatings using site-selective bioconjugation. J. Am. Chem. Soc. 2010; 132: 13264–13269. 10.1021/ja103038p 20825180

[13] Kim M, Gwak Y, Jung W, Jin ES. Identification and Characterization of an Isoform Antifreeze Protein from the Antarctic Marine Diatom, Chaetoceros neogracile and Suggestion of the Core Region. Mar. Drugs. 2017; 15: no. 318, 1–14. 10.3390/md15100318 PMC5666426 29057803

[14] Gwak Y, Park J, Kim M, Kim HS, Kwon MJ, Oh SJ et al Creating anti-icing surfaces via the direct immobilization of antifreeze proteins on aluminum. Sci. Rep. 2015; 5: no. 12019, 1–9. 10.1038/srep12019 PMC4495550 26153855

[15] Charpentier TVJ, Neville A, Millner P, Hewson R, Morina A. An investigation of freezing of supercooled water on anti-freeze protein modified surfaces. J. Bionic Eng. 2013; 10: 139–147.

[16] Miyamoto T, Nishi N, Waku T, Tanaka N, Hagiwara Y. Effects of short-time preheating on ice growth in antifreeze polypeptides solutions in a narrow space, Heat and Mass Transfer. 2018; 54: 2415–2424.

[17] Marshall CB, Chakrabartty A, Davies PL. Hyperactive antifreeze protein from winter flounder is a very long rod-like dimer of α-helices. J. Bio. Chem. 2005; 280: 17920–17929. 15716269 10.1074/jbc.M500622200

[18] Evans RP, Fletcher GL. Isolation and characterization of type I antifreeze proteins from Atlantic snailfish (Liparis atlanticus) and dusky snailfish (Liparis gibbus). Biochimica et Biophysica Acta. 2001; 1547: 235–244. 11410279 10.1016/s0167-4838(01)00190-x

[19] Kun H, Mastai Y. Activity of short segments of type I antifreeze protein. Peptide Science. 2007; 88: 807–814. 10.1002/bip.20844 17868093

[20] Nishi N, Miyamoto T, Waku T, Tanaka N, Hagiwara Y. Ice growth inhibition in antifreeze polypeptide solution by short-time solution preheating. PLOS ONE 2016; 11: article no. 0154782, 1–15. 10.1371/journal.pone.0154782 PMC4859470 27152720

[21] Nishi N. Creation of functional anti-icing surfaces by applying antifreeze protein and protein immobilization technology (in Japanese). Master thesis, Kyoto Institute of Technology, 2016.

[22] Chaudhary G, Li R. Freezing of water droplets on solid surfaces: An experimental and numerical study. Exp. Therm. and Fluid Sci. 2014; 57: 86–93.

[23] Graeber G, Schutzius TM, Eghlidi H, Poulikakos D. Spontaneous self-dislodging of freezing water droplets and the role of wettability, Proc. Natl Acad. Sci. USA 2017; 114: 11040–11045. 10.1073/pnas.1705952114 28973877 PMC5651746

[24] Kim P, Wong T-S, Alvarenga J, Kreder MJ, Adorno-Martinez WE, Aizenberg J. Liquid-infused nanostructured surfaces with extreme anti-ice and anti-frost performance. ACS Nano 2012; 6: 6569–6577. 10.1021/nn302310q 22680067

[25] Wang Y, Yao X, Chen J, He Z, Liu J, Li Q et al Organogel as durable anti-icing coatings. Sci. China Mater. 2015; 58: 559–565.

[26] Chen J, Luo Z, Fan Q, Lv J, Wang J. Anti-ice coating inspired by ice skating. Small 2014; 10: 4693–4699. 10.1002/smll.201401557 25145961

[27] Chernyy S, M, Shimizu K, Swerin A, Pedersen SU, Daasbjerg K et al Superhydrophilic polyelectrolyte brush layers with imparted anti-icing properties: effect of counter ions. ACS Appl. Mater. Interfaces 2014; 6: 6487–6496. 10.1021/am500046d 24713022

[28] Meuler AJ, Smith JD, Varanasi KK, Mabry JM, McKinley GH, Cohen RE. Relationships between water wettability and ice adhesion. ACS Appl. Mater. Interfaces 2010; 2: 3100–3110. 10.1021/am1006035 20949900

[29] Yin X, Zhang Y, Wang D, Liu Z, Liu Y, Pei X et al Integration of self-lubrication and near-infrared photothermogenesis for excellent anti-icing/deicing performance. Adv. Funct. Mater. 2015; 25: 4237–4245.

[30] Matsumoto K, Kobayashi T. Fundamental study on adhesion of ice to cooling solid surface. Int. J. Refrig. 2007; 30: 851–860.

[31] Oberli L, Caruso D, Hall C, Fabretto M, Murphy PJ, Evans D. Condensation and freezing of droplets on superhydrophobic surfaces. Adv. in Colloid and Interface Science 2014; 210: 47–57. 10.1016/j.cis.2013.10.018 24200089

[32] Agui H, Yonezawa S, Ohkubo H, Hagiwara Y. Frost formation on micro-grooved glass surfaces. In preparation for submission.

[33] Hayakari K, Hagiwara Y. Effects of ions on winter flounder antifreeze protein and water molecules near an ice/water interface. Molecular Simulation 2012; 38: 26–37.

[34] Nakamura M, Fushida M, Okuda S. Formation of ice on several substrates (in Japanese). J. Ceramic Assoc. Japan. 1986; 94: 571–576.

