# Supplementary figures and images for: Suppression of droplets freezing on glass surfaces on which antifreeze polypeptides are adhered by a silane coupling agent
Source: PLoS One. 2018 Oct 5;13(10):e0204686. doi: 10.1371/journal.pone.0204686 (PMC6173376; doi:10.1371/journal.pone.0204686)

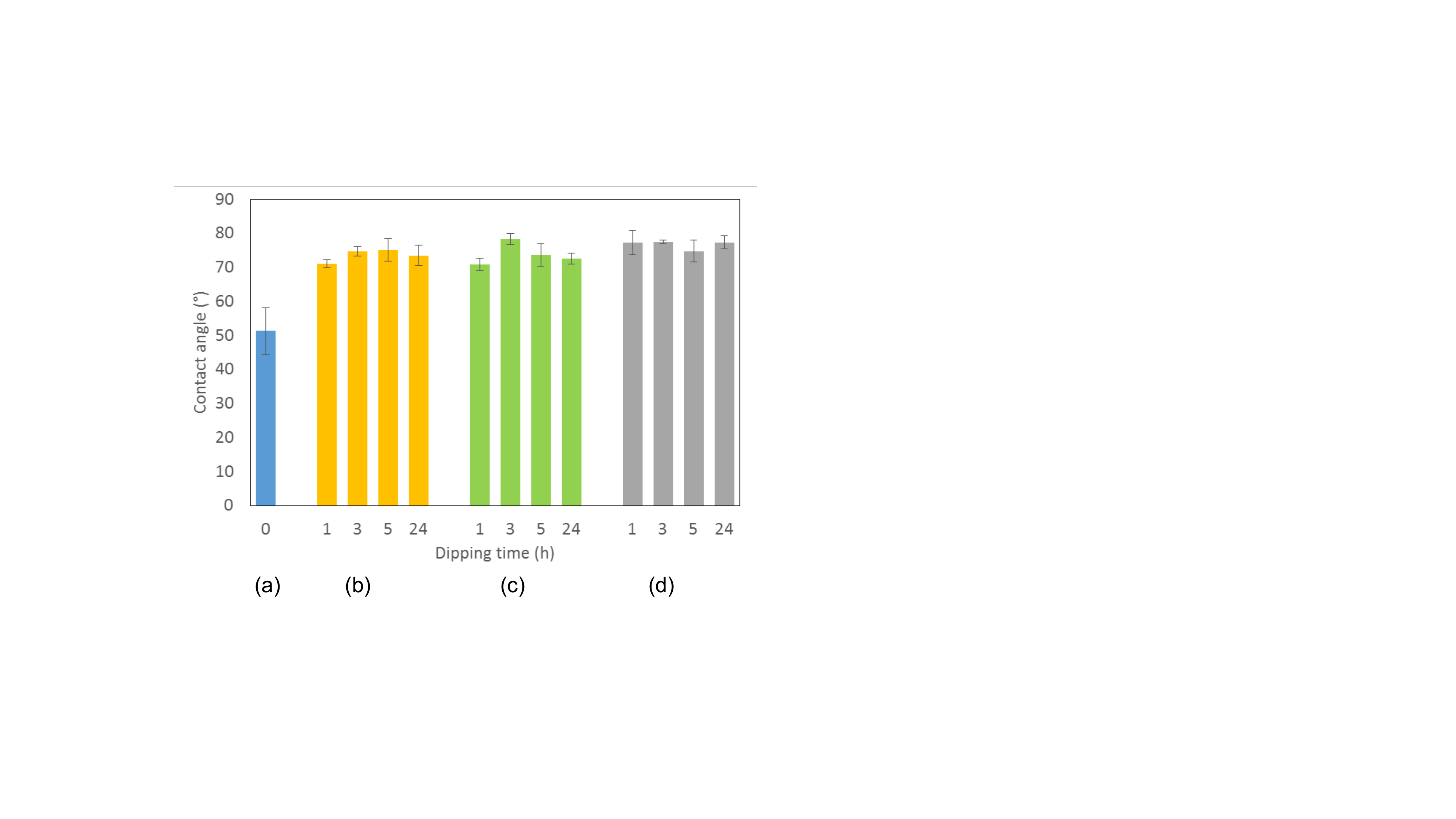

Supplement: S1 Fig — (a) unprocessed glass plates, (b) in the case where the concentrations of APTMS and ethanol were 1 wt%, (c) in the case where the concentrations of APTMS and ethanol were 2 wt% and 1 wt%, respectively, and (d) in the case where the concentrations of APTMS and ethanol were 2 wt%. (TIF) [file pone.0204686.s001.tif]
